# Factors affecting access to information on routine immunization among mothers of under 5 children in Kaduna State Nigeria, 2015

**DOI:** 10.11604/pamj.2017.27.186.11191

**Published:** 2017-07-10

**Authors:** Lydia Taiwo, Suleiman Idris, Aisha Abubakar, Patrick Nguku, Peter Nsubuga, Saheed Gidado, Lilian Okeke, Samuel Emiasegen, Endie Waziri

**Affiliations:** 1 Nigeria Field Epidemiology & Laboratory Training Program (NFELTP), Abuja, Nigeria; 2 Federal Ministry of Health, Abuja, FCT, Nigeria; 3 Department of Community Medicine, Ahmadu Bello University, Zaria, Nigeria; 4 Global Public Health Solutions, Atlanta GA, USA

**Keywords:** Routine immunization, information, mothers, caregivers

## Abstract

**Introduction:**

Immunization is one of the most effective interventions to prevent disease and early child death. A substantial number of children worldwide do not complete immunization schedules because neither health services nor conventional communication mechanisms regularly reach their communities. Knowledge and perception of mothers/caregivers regarding VPDs influence demand and utilization of immunization services. We examined the associations between knowledge, perception and information on routine immunization received by mothers/caregivers in Kaduna State.

**Methods:**

We enrolled 379 eligible caregivers in a community-based cross-sectional study. We sampled respondents using multistage sampling technique. We collected data on socio-demographic characteristics; knowledge and perception on routine immunization using semi-structured interviewer-administered questionnaire. We conducted bivariate analysis and logistic regression using Epi-InfoTM version 7 at 5% level of significance.

**Results:**

Mean age of respondents was 28.6 years (standard deviation=±6.6 years), 34% completed secondary school, 65% were unemployed, 49% lived in rural settlements. Among respondents' children 53.3% were females and 62.8% fell within 2^nd^-5^th^ birth order. Only 15.6% of these children were fully immunized. Seventy-five percent of respondent did not obtain information on routine immunization within 12 months prior to the study. About 64% had unsatisfactory knowledge while 55.4% exhibited poor perceptions regarding routine immunization. Commonest source of information was radio (61.61%). On logistic regression educated participants (Adjusted odds ratio (AOR)=1.9, 95% CI: 1.1-3.3), mothers' perception (AOR=2.6, 95% CI: 1.5-4.5) and monogamous family setting (AOR=2.4, 95% CI: 0.2-0.6) were likely to have obtained information on routine immunization.

**Conclusion:**

There is low access to information, poor maternal knowledge on routine immunization with low vaccination coverage in this community. Efforts should be made by the Governments to scale up sensitization of mothers/caregivers to improve their knowledge on routine immunization through radio jingles.

## Introduction

Immunization is one of the safest and most effective interventions to prevent disease and early child death [1]. Although three-quarters of the world's child population is reached with the required vaccines, only half of the children in sub-Saharan Africa get access to basic immunization [2]. A substantial number of children worldwide do not complete immunization schedules because neither health services nor conventional communication mechanisms regularly reach their communities [3]. Separate studies in Australia and Papua New Guinea have shown that knowledge gaps underlie low compliance with vaccination schedules [4, 5]. Mothers or caregivers are less likely to complete immunization schedules if they are poorly informed about the need for immunization, logistics (which includes time, date and place of vaccination) and the appropriate series of vaccines to be followed [5, 6]. Although knowledge in itself is insufficient to create demand, poor knowledge about the need for vaccination and when the next vaccination is due is a good indicator of poor compliance [7]. Up-to-date, complete and scientifically valid information about vaccines can help parents to make informed decisions [8]. Only 10% of children in the North West Nigeria are fully immunized, compared to 52% of children in the South East and South West geopolitical zones despite the numerous vaccination campaigns in the area [9].

A separate study reported that the low immunization coverage in North West Nigeria, may be due to social and cultural practices that restrict most women from obtaining basic information on immunization in the [10]. The low routine immunization coverage in Kaduna state has created an immunity gap which favors the emergence and transmission of vaccine-preventable diseases (VPD) like measles and polio [9]. According to the World Health Organization (WHO), the persistence of VPD in Nigeria can largely be attributed to the under-utilization of available vaccines [11]. Knowledge gaps by the mother or caregivers may lead to low compliance with vaccination schedules. Also, some health centers are situated far from the communities and this may not allow mothers to complete their children's immunization schedules because neither health services nor conventional communication mechanisms regularly reaches these communities; which is the case in North West Nigeria [1]. Tracking, evaluating and assessing the type and quality of information received by mothers provides a vital tool for understanding routine immunization gaps in Kaduna State. We decided to examine the associations between knowledge, perception and information on routine immunization received by mothers or caregivers in Kaduna State, to guide the government make an informed decision on improving their vaccination coverage.

## Methods

**Study area**: Kaduna State, the third most populous state in Nigeria, is in the North West geographical zone and shares boundaries with Niger State to the West, Zamfara, Katsina and Kano States to the North, Bauchi and Plateau State to the East, and Federal Capital Territory (FCT) and Nasarawa State to South. The state is culturally diverse with a projected population of 7,589,699 people (projection from 2006 Census). The state is divided into three Senatorial zones and has 23 Local Government Areas (LGAs) with rural and semi-urban settlements in most of the LGAs. Kaduna State has five tertiary hospitals, 28 secondary hospitals, >1,000 primary health care facilities (PHCs) and an estimated 656 private facilities with under 5 year old population of about 1,536,988 [12]. The majority of the Government-owned facilities conduct facility based sessions on routine immunization and outreaches to the hard to reach areas.

**Study population, design, and sampling**: The study population was mothers or caregivers whose children were within the age group 12-23 months in Kaduna State who consented. Mothers or caregivers that were too sick to participate or not available at the time of interview were excluded from the study. We conducted a cross-sectional study between April and June 2015 with minimum sample size required for the study calculated by using the formula for single proportions and based on an estimate of 34% which is the proportion of mothers with knowledge on immunization from a study conducted in Zamfara State, standard normal deviate set at 1.96 (for 95% confidence level), and precision of 0.05.

$$n=\frac{z_{\alpha }{}^{2}pq}{d^{2}}$$

The calculated minimum sample size was 345. Adjusting for non-response and missing data of 10% gave a minimum sample size of 379 respondents. We used a multi-stage sampling technique using the three senatorial zones in Kaduna State. Kaduna State was stratified into three (according to senatorial zones) and two LGAs (Rural and Semi-urban LGA) were selected from each stratum using simple random sampling. Two wards were selected from each of the six LGAs using simple random sampling. Two settlements were selected from each of the 12 wards using simple random sampling and finally we administered 16 questionnaires to contiguous households in each of the selected settlements after spinning a bottle to identify the first household.

**Data collection methods**: We collected data using a pre-tested, interviewer-administered questionnaire, which had five sections: socio-demographic factors; sources of information on routine immunization; child's immunization record, reasons for not immunizing children and knowledge and perception of mothers or caregivers on immunization. Having selected the study LGAs, the survey instrument was pre-tested in Zaria and questions found to be unclear or unnecessary were modified or deleted accordingly. Appropriate corrections were captured subsequently to establish validity and reliability. Twelve research assistants, two per LGA with minimum health qualification community health extension worker, were recruited and trained to standardize data collection procedures for the purpose of this study.

**Data management and analysis**: We entered data, cleaned and analyzed using Epi InfoTM version 7 (US centers ford disease control and prevention). We performed descriptive statistics using absolute numbers for univariate for simple percentages, range, and measures of central tendency, bivariate analysis where the odds ratio and chi-square test were determined between variables and p≤0.05 was considered statistically significant and lastly multivariate analysis was conducted using logistic regression.

**Method of grading knowledge and perception of respondents**: A total of seven variables were assessed for knowledge with a score range of 0-21. Respondents who scored 10-21 were classified to have satisfactory knowledge while those with scores of 0-9 had unsatisfactory knowledge. Similarly, 11 variables were assessed for practices with a score range of 0-33. Respondents with scores of 16-21 were classified to have perception while those with scores of 15 and less had poor perception [10].

**Ethical considerations**: We obtained ethical clearance from the Kaduna State Research Ethics Committee, Kaduna state Ministry of Health. A verbal and written consent was obtained from the respondents.

## Results

**Socio-demographic factors of respondents**: A total of 379 respondents were recruited with a mean age of 28.6 years standard deviation (SD)=±6.6 years), 245 (64.7%) practiced Islam, 128 (33.8%) completed secondary school, 246 (64.9%) were unemployed, 361 (92.3%) were married and 186 (49.1%) lived in rural settlements (Table 1).

**Table 1:** Socio-demographic characteristics of mothers in Kaduna State Nigeria, 2015

Characteristics	Frequency	Percent (%)
**Age group (years)**		
15-24	31	8.2
20-24	83	21.9
25-29	91	24.0
30-34	95	25.1
35-39	49	12.9
≥40	30	7.9
**Mean age 28.6 SD ± 6.6 years**		
**Religion**		
Christianity	134	35.4
Islam	245	64.6
**Educational level**		
Primary	67	17.7
Secondary	48	12.7
Tertiary	80	21.0
Koranic	159	42.0
None	25	6.6
**Occupation**		
Unemployed	246	64.9
Trading	69	18.2
Farming	22	5.8
Employer	42	11.1
**Marital status**		
Single	6	1.6
Married	361	92.2
Divorced	9	2.4
Widowed	1	0.3
Separated	2	0.5
**Family setting**		
Polygamous	234	65.8
Monogamous	127	35.2
**Settlement type**		
Semi-urban	193	50.9
Rural	186	40.1

Majority of respondents were unemployed, married women in polygamous setting

**Knowledge and perception as regards routine immunization and vaccine preventable diseases**: A total of 246 (65%) respondents knew vaccination protects against VPD and should be completed within 12 months of age, 18 (4.8%) knew the correct route of administering the vaccine and 25 (6.6%) knew that VPDs cause severe disability and death. The commonest symptoms of VPD that were mentioned by the respondents were fever, mentioned by 281 (74.2%) and cough mentioned by 203 (53.6%). While 273 (72%) of respondents said their problem is malaria and not immunization, 167 (44%) said that going for immunization is a waste of time, 91 (24%) believed only sick children take immunization, 310 (82%) said getting multiple shots in one visit can overload a child's system, and 190 (50%) said vaccination causes infertility later in adult life.

**Grading of knowledge and perception**: A higher proportion of respondents 244 (64.4%) had unsatisfactory knowledge and 197 (55.4%) exhibited poor perception regarding routine immunization (Table 2).

**Table 2:** Graded knowledge and perception of mothers on routine immunization in Kaduna State Nigeria, 2015

Variable	Frequency	Percent
**Knowledge (n=379)**		
Satisfactory knowledge	135	35.6
Unsatisfactory knowledge	244	64.4
**Perception (n=372)**		
Good perception	175	44.6
Poor perception	197	55.5

Majority of respondents had unsatisfactory knowledge with poor perceptions on routine immunization

**Information received on routine immunization**: A total of 267 (70.5%) of the respondents reported that they did not get information on routine immunization within 12 months before the study. Out of those that had information, 83 (74%) reported that they had received information about the benefits of routine immunization with the source of information being radio 69 (61.6%) (Table 3).

**Table 3:** Information received by mothers on routine immunization within 12 months prior to study in Kaduna State, Nigeria-2015

Characteristics	Frequency	Percent
**Received information on RI within the past 12months**		
Yes	112	29.6
No	267	70.4
**Types of information received on routine immunization**		
Benefits of routine immunization	83	74.0
Schedule of routine immunization	15	13.4
Side effects of routine immunization	4	3.6
Cannot remember any	10	9.0
**Main source of information on routine immunization**		
Health Facility	23	20.5
Radio	69	61.6
Television	9	8.0
Newspaper	1	0.9
Mosque	2	1.8
Church	3	2.7
Community meetings	5	4.5

Less than 30% of respondents had access to information on routine immunization within 12 months prior to the study

**Vaccination coverage**: Fifty-nine (15.6%) of respondents had fully immunized their children as evidenced by vaccination card history (Table 4). Respondents who vaccinated their children were six times more likely to have received information on routine immunization within 12 months before the study than those who did not vaccinate their children (odds odds ratio (OR)=6.19 95% confidence interval (CI)(3.60-11.50).

**Table 4:** Vaccination status of children studied based on immunization card and mothers' recall in Kaduna State, Nigeria-2015

Vaccination status	Mothers recall n (%)	Immunization card n (%)
Unimmunized	112 (29.6)	202 (53.3)
Partially immunized	121 (31.9)	118 (31.1)
Fully immunized	146 (38.5)	59 (15.6)

Only 15% of respondents were found to have completed their children’s vaccination as evidenced by child’s immunization cards

**Figure 1:**
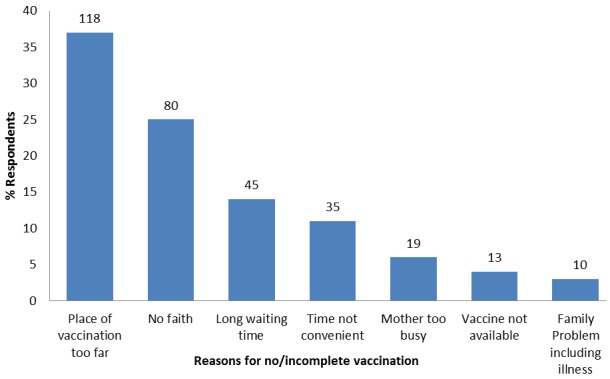
Reasons why respondents did not immunize or complete their children's vaccination in Kaduna State, Nigeria-2015

**Reasons for no or incomplete vaccination**: Among the 320 respondents who did not vaccinate or complete their child's vaccination, 118 (37%) of them said the place of immunization was too far, 80 (25%) had no faith that immunization will protect their children against vaccine-preventable diseases. Thirteen (4%) of the respondents did not complete their child's vaccination because the vaccine was not available (**Figure 1**).

**Determinants of access to information on routine immunization**: In the multivariate analysis after adjusting for age, educational status, employment status, family setting, settlement type, knowledge and perception of respondents on routine immunization, mothers with education were twice more likely to have obtained information on routine immunization (95% CI: 1.1-3.3). Mothers with good perception were 2.6 times more likely to have obtained information on routine immunization (95% CI: 1.5-4.5) and those in monogamous family setting were less likely to have to have obtained information on routine immunization (adjusted OR 0.6 95% CI: 0.2-0.6). Mothers with unsatisfactory knowledge were less likely to have to have obtained information on routine immunization (adjusted OR 0.3 95% CI: 0.2-0.7) (Table 5).

**Table 5:** Determinants of respondents' information on routine immunization and selected variables in Kaduna State, Nigeria-2015

Variables	Bivariate analyses Received information on RI			Odds ratio	Multivariate Analyses (Unconditional logistic regression)	
	Yes n(%)	No n(%)	Total n(%)		AOR*	95% CI
*Age(Yrs)*						
>28	44(23.3)	145(76.7)	189(100.0)	1.84 (1.17-2.87) †	1.56	0.97-2.51 †
≤28	68(35.8)	122(64.2)	190(100.0)			
**Educational Status**						
Educated	67(52.3)	61(47.7)	128(100.0)	3.42 (2.11-3.67) †	1.90	1.11-3.28††
Not Educated	45(17.9)	206(82.1)	251(100.0)			
Employment Status						
Not Employed	107(31.8)	230(68.3)	337(100.0)	0.29 (0.11-0.76) †	0.35	0.89-6.40
Employed	5(11.9)	37(88.1)	42(100.0)			
**Family Setting**						
Monogamous	45(22.5)	155(77.5)	200(100.0)	2.04 (1.29-3.21) †	2.39	0.20-0.59††
Polygamous	60(37.3)	101(62.7)	161(100.0)			
**Settlement Type**						
Semi-urban	46(28.8)	147(76.2)	193(100.0)	1.76 (1.12-2.75) †	1.55	0.96-2.49
Rural	66(35.5)	120(64.5)	186(100.0)			
**Knowledge on RI**						
Unsatisfactory	92(33.7)	152(62.3)	244(100.0)	0.26 (0.15-0.50) †	0.33	0.20-0.56††
Satisfactory	20(14.8)	115(85.2)	135(100.0)			
**Perceptions**						
Good	43(24.6)	135(75.4)	175(100.0)	2.55 (1.60-4.05) †	2.60	1.50-4.51††
Poor	66(33.5)	131(66.5)	197(100.0)			

* Adjusted odds ratio (AOR)

† Significant at bivariate analysis; variables that were significant in bivariate analysis were included in logistic regression model

†† Significant at multivariate analysis

## Discussion

Our study found that majority of mothers had not obtained information on and possessed poor knowledge on routine immunization in Kaduna State Nigeria. Lack of access to information on routine immunization could have been the reason for their poor knowledge and perception. It also could have resulted in the low immunization coverage and high dropout rate as we found that mothers who received information on routine immunization and VPD were likely to have vaccinated their children. Our finding is similar to separate studies carried out in Zamfara, Nigeria and Turkey. The majority of respondents had poor knowledge on routine immunization and this was found to be associated with not getting information on routine immunization and subsequently not vaccinating children. Similar to findings were seen in studies where mother's knowledge about immunization was found to be a predictor of full immunization in urban and rural areas of Nigeria [10, 11, 13]. Our study found that majority of respondents knew immunization protects children against VPDs (66.8%), and basic childhood immunization should be completed within 12 months of age (88.5%). Four percent (4.2%) of the respondents knew the various routes of administration of vaccines and (6.6%) knew that VPD can cause serious disability and even death. Most of the respondents knew that fever (74.2%) and cough (53.6%) is the commonest symptoms of VPDs. Similar findings were found in a study conducted in Zamfara State, [10]. We found that the majority (62%) of respondents who obtained information on routine immunization acquired it from the radio, possibly because a significant proportion (52.8%) of respondents are rural dwellers who most often than not rely on radio as a means of getting information concerning the outside world.

Our study found that their source of information influenced their likelihood of higher participation in getting information on routine immunization (OR= 1.76, CI=1.1-2.8) than the semi-urban respondent. Awareness through the radio and television have increased vaccination rate in Mexico and Bangladesh where mass media is accessible and widely accessed as seen in two separate studies [14, 15]. Our finding is however not consistent with a study conducted in southwest Nigeria, which showed that 65% of women got information on routine immunization at the antenatal clinics (ANC) [13]. We also found that women older than 28 years had a higher likelihood (OR=1.84) of getting information on routine immunization than the those that were less than 28 years old, similar to findings from studies conducted in Sudan and Ibadan [16, 17]. Our study showed that two-thirds of the mothers and caregivers in Kaduna State had no formal education, this possibly contributed to the low literacy level of women in the North West Nigeria. This finding was consistent with a study in Togo [18]. Our study revealed that maternal education and good perception were independently associated with access to information on routine immunization and mothers from polygamous family setting, and those with poor knowledge did not get information on routine information and could possibly not have immunized their children. These findings are in consonant with other studies [19–22]. Our study was limited by some factors. First, there was a possibility of recall bias by respondents. We did not explore other factors such as level of knowledge and perception of health care personnel on routine immunization, especially because they play important roles in immunization activities. Secondly, we did not find out male involvement in their children's vaccination. The questions in the data tool were however framed and translated to capture all in the best possible way to avoid recall bias and give insight into the roles of husbands in child immunization.

## Conclusion

There was low access to information and poor maternal knowledge on routine immunization with low vaccination coverage in this community. Kaduna state government, through the Ministry of Information, should intensify sensitization of mothers or caregivers to improve their knowledge on routine immunization through radio jingles. Local government authorities should provide parents with basic health information on routine immunization by distributing printed materials such as brochures, pamphlets and leaflets in local languages.

### What is known about this topic

- A substantial number of children worldwide do not complete immunization schedules because neither health services nor conventional communication mechanisms regularly reach their communities;
- Mothers or caregivers are less likely to complete immunization schedules if they are poorly informed about the need for immunization, logistics (which includes time, date and place of vaccination) and the appropriate series of vaccines to be followed;
- Up-to-date, complete and scientifically valid information about vaccines can help parents to make informed decisions.

### What this study adds

- Majority of caregivers in Kaduna State Nigeria had poor access to information on routine immunization;
- Only fifty-nine (15.6%) of respondents had fully immunized their children as evidenced by vaccination card history;
- The most common source of information on routine immunization was through radio jingles.

## Competing interests

The authors declare no competing interests.
